# Specificity and Dynamics of Effector and Memory CD8 T Cell Responses in Human Tick-Borne Encephalitis Virus Infection

**DOI:** 10.1371/journal.ppat.1004622

**Published:** 2015-01-22

**Authors:** Kim Blom, Monika Braun, Jolita Pakalniene, Laura Dailidyte, Vivien Béziat, Margit H. Lampen, Jonas Klingström, Nina Lagerqvist, Torbjörn Kjerstadius, Jakob Michaëlsson, Lars Lindquist, Hans-Gustaf Ljunggren, Johan K. Sandberg, Aukse Mickiene, Sara Gredmark-Russ

**Affiliations:** 1 Center for Infectious Medicine, Department of Medicine, Karolinska Institutet, Karolinska University Hospital Huddinge, Stockholm, Sweden; 2 Department of Infectious Diseases, Lithuanian University of Health Sciences, Kaunas, Lithuania; 3 Human Genetics of Infectious Diseases Laboratory, Imagine Institute—INSERM U1163, Paris, France; 4 Karolinska University Laboratory, Department of Clinical Microbiology, Karolinska University Hospital Solna, Stockholm, Sweden; 5 Department of Infectious Diseases, Karolinska University Hospital Huddinge, Stockholm, Sweden; 6 Unit of Infectious Disease, Department of Medicine, Karolinska Institutet, Karolinska University Hospital Huddinge, Stockholm, Sweden; University of Pennsylvania, UNITED STATES

## Abstract

Tick-borne encephalitis virus (TBEV) is transferred to humans by ticks. The virus causes tick-borne encephalitis (TBE) with symptoms such as meningitis and meningoencephalitis. About one third of the patients suffer from long-lasting sequelae after clearance of the infection. Studies of the immune response during TBEV-infection are essential to the understanding of host responses to TBEV-infection and for the development of therapeutics. Here, we studied in detail the primary CD8 T cell response to TBEV in patients with acute TBE. Peripheral blood CD8 T cells mounted a considerable response to TBEV-infection as assessed by Ki67 and CD38 co-expression. These activated cells showed a CD45RA-CCR7-CD127- phenotype at day 7 after hospitalization, phenotypically defining them as effector cells. An immunodominant HLA-A2-restricted TBEV epitope was identified and utilized to study the characteristics and temporal dynamics of the antigen-specific response. The functional profile of TBEV-specific CD8 T cells was dominated by variants of mono-functional cells as the effector response matured. Antigen-specific CD8 T cells predominantly displayed a distinct Eomes+Ki67+T-bet+ effector phenotype at the peak of the response, which transitioned to an Eomes-Ki67-T-bet+ phenotype as the infection resolved and memory was established. These transcription factors thus characterize and discriminate stages of the antigen-specific T cell response during acute TBEV-infection. Altogether, CD8 T cells responded strongly to acute TBEV infection and passed through an effector phase, prior to gradual differentiation into memory cells with distinct transcription factor expression-patterns throughout the different phases.

## Author Summary

Tick-borne encephalitis virus (TBEV) belongs to the flavivirus family and causes tick-borne encephalitis. This is a severe meningoencephalitic disease with no available treatment. Detailed studies of the immune response during human TBEV infection are essential to understand host responses to TBE and for the development of therapeutics. Herein, we studied the primary T cell-mediated immune response in patients diagnosed with TBEV infection. We show that CD8 T cells mount a vigorous TBEV-specific response within one week of hospitalization. Moreover, TBEV-specific CD8 T cells displayed a distinctive phenotypic and functional profile, paired with a distinct transcription factor expression-pattern during the peak of activation. In summary, this is the first comprehensive study of the CD8 T cell response during acute human TBEV infection, and provides a framework for understanding of CD8 T cell-mediated immunity in this emerging viral disease.

## Introduction

Tick-borne encephalitis virus (TBEV) is a single-stranded flavivirus and the causative agent of tick-borne encephalitis (TBE). TBEV is transferred to humans from infected Ixodes ticks. TBE is an increasing public health problem occuring throughout northern and central Europe and Asia, with thousands of encephalitis cases reported annually despite available TBE vaccines [1, 2]. Epidemiological studies suggest that around 25% of all infected individuals develop clinical disease [3, 4]. TBE has a characteristic biphasic course with influenza-like illness followed by a second neuroinvasive phase with neurological symptoms of variable severity, ranging from meningitis to severe meningoencephalitis. About a third of the patients eventually suffer from long-term sequelae including neuropsychiatric problems, headaches, and a substantial decrease in quality of life (reviewed in [5]).

The mechanisms behind TBE-pathogenesis are largely unknown. Direct infection of neurons has been suggested as the cause of neurological disease, and TBEV is present in brain tissue in most of the fatal cases [6]. More severe disease has been associated with low levels of neutralizing antibodies to TBEV, as well as a low early cerebrospinal fluid (CSF) IgM response [7]. The few studies that have addressed T cell responses in TBE have suggested that immunopathological effects caused by T cells may influence disease outcome, based on data showing CD8 T cell infiltration in brain tissue in fatal cases [8]. Murine models support this notion, demonstrating a prolonged survival of CD8-deficient and SCID mice, as compared to immunocompetent mice, following experimental viral infection [9]. In parallel, clonal T cell infiltration has been observed in the brains of mice dying from TBE [10].

Most current knowledge of antigen-specific CD8 T cell responses to acute primary viral infections still comes from murine models, where responses to viruses such as lymphocytic choriomeningitis virus (LCMV) or vaccinia virus have been studied [11, 12]. In such models, activated T cells undergo a phase of rapid proliferation with an expansion of Ag-specific CD8 T cell clones. During the peak response, a majority of all CD8 T cells may be specific for the infecting virus. The response then contracts, forming a smaller memory population following clearance of the virus [11, 13]. Human antiviral CD8 T cell responses have also been extensively analyzed in chronic infections, such as in infections with human immunodeficiency virus (HIV), cytomegalovirus (CMV) and Epstein-Barr virus (EBV) [14–17]. Based on the results from such studies, distinct stages of CD8 T cell differentiation have been defined by the expression of specific surface markers, such as the isoforms of CD45 and of the expression of homing receptor CCR7, defining CD45RA^+^CCR7^+^ as naïve, CD45RA^−^CCR7^+^ as central memory (Tcm), CD45RA^−^CCR7^−^ as effector memory (Tem), and CD45RA^+^CCR7^−^ as effector memory RA (TemRA) CD8 T cells [18, 19].

Recent studies, adopting the live attenuated yellow fever virus (YFV) vaccine as a model to study acute viral infection in humans, have indicated that CD8 T cells against HLA-A2- and HLA-B7-restricted epitopes display a CD45RA^−^CCR7^−^ phenotype during the peak effector response, which transits into a CD45RA^+^CCR7^−^ phenotype at the memory stage [20, 21]. Studies from the YFV and smallpox vaccine model systems have suggested that the magnitude of the total effector CD8 T cell response can be quantified with a set of four phenotypic markers, with the transient Ki67^+^Bcl-2^low^HLA-DR^+^CD38^+^ phenotype defining the effector CD8 T cells during acute viral infection [22].

In recent years, the T-box transcription factors T-bet and Eomesodermin (Eomes) have been shown to play important roles in determining the fate of murine CD8 T cells during infection [23–25]. Their cooperative expression in chronic infection has been shown to be critical to sustain viral control since deletion of either one of them resulted in failure to control infection [26]. The expression patterns of T-bet and Eomes in CD8 T cells is not yet completely understood, and the analysis of these transcription factors during CD8 T cell differentiation may bring a novel molecular perspective to the phenotypic characterization of CD8 T cells and a deeper understanding of CD8 T cell differentiation during both acute and chronic viral infections.

In this study, we have investigated the human CD8 T cell response to TBEV in a cohort of infected patients. The activated CD8 T cells were characterized with respect to perforin, granzyme B, HLA-DR, PD-1, T-bet and Eomes and Bcl-2 expression. An immunodominant HLA-A2-restricted TBEV epitope was identified, which allowed for a detailed analysis of the antigen-specific CD8 T cell response. Studying these parameters with the tools available, the present study characterizes the kinetics and characteristics of the CD8 T cell response to TBEV. The implications of the findings are discussed in the context of host response to TBEV, possible role in immunopathogenesis, therapy and vaccination.

## Results

### Study cohort and characteristics

Peripheral blood mononuclear cells (PBMCs), cerebrospinal fluid (CSF), sera, and whole blood were collected from 20 patients with confirmed TBE (IgM positive for TBEV in serum according to standard clinical diagnostic criteria) (S1 Table). Blood samples were obtained within 3 days after hospitalization (day 0) and after 7, 21, and 90 days, during an interval ranging from the acute neuroinvasive phase to the convalescent phase of disease. Patients were evaluated at all sampling time points for symptoms such as fever, headaches, dizziness, impaired consciousness, nuchial rigidity, tremor, nystagmus, ataxia, emotional lability, difficulty in concentration, impaired memory, dysphasia, dysaesthesia, sleeping disturbances, irritability toward light and/or sound, generalized seizures, alterations of reflexes, impaired hearing, and disturbances of the cranial or spinal nerves. Patients were classified as having mild (meningitis) (n = 16) or moderate (meningoencephalitis) (n = 4) forms of TBE according to previous definitions [27]. Negative control samples were collected from 20 age-matched, healthy control subjects who were not previously vaccinated against TBE or had no symptoms of clinical TBE infection. Median time since symptom debut in the secondary neuroinvasive phase was 4 days (range 1–15), whereas the median time since symptom debut in the first phase were 15 days (range 5–24), at the time of inclusion. All patients had seroconverted at the time of inclusion. While all the patients were positive for TBEV IgM at day 0 and 7 after hospitalization (S1 Table), all but one patient were positive for IgG at day 0, and all donors had increasing levels of IgG over time (S1 Fig.). TBEV RNA was not detected in plasma or CSF from any individual.

### Identification and characterization of TBEV-induced effector T cells in peripheral blood

**Figure 1:**
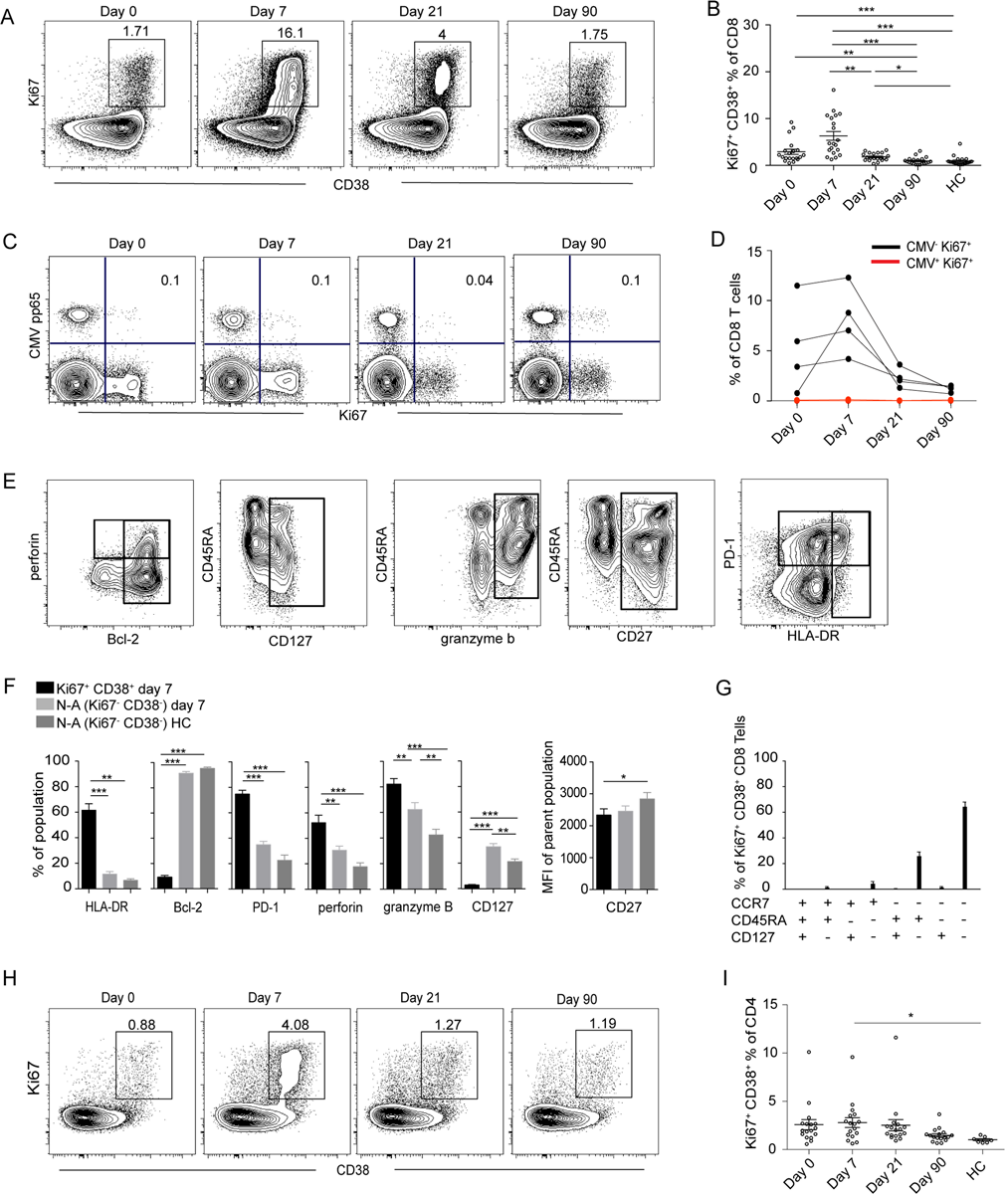
Activation of T cells in the acute phase of TBE infection. (A) CD38 and Ki67 co-expressing cells in the total CD8 T cell population over time in one representative patient. (B) Median and 10–90th percentiles of CD38 and Ki67 co-expression in CD8 T cell subsets at day 0, 7, 21 and 90 after hospitalization in infected subjects (n = 20) and in healthy controls (n = 20). (C) Ki67 expression vs CMV-pp65 HLA MHC class I tetramer staining over time in one donor. Percent Ki67^+^ CMV pp65^+^ cells are indicated in the plot. (D) Kinetics of Ki67 expression in CMV^+^ (red line) and CMV^−^ (black line) CD8 T cells in four donors over time. (E) Stainings of perforin, CD45RA, PD-1, Bcl-2, CD127, granzyme B, CD27 and HLA-DR at day 7 after hospitalization. Gated on total CD8 T cells. (F) Bar plots show the 10–90th percentiles of HLA-DR, Bcl-2, PD-1, granzyme B, perforin and CD127 expression together with CD27 in terms of mean fluorescence intensity in CD38 and Ki67 co-expressing CD8 T cell subset at day 7 after hospitalization, non-activated Ki67^−^CD38^−^ (N-A) cells at day 7 after hospitalization or in non-activated healthy controls (N-A HC). (G) Bar chart represents the subset distribution of CCR7, CD45RA and CD127 (IL7Rα) in CD38 and Ki67 co-expressing cells at day 7 after hospitalization. (H) CD38 and Ki67-coexpressing cells in CD4 cell population over time in one infected patient. (I) Median and 10–90^th^ percentiles of CD38 and Ki67 co-expression in CD4 T cell subset at the day of hospitalization (day 0) and at day 7, 21 and 90 after hospitalization in infected subjects (n = 20) together with healthy controls (n = 20). Statistical analysis was performed using non-parametric repeated measures ANOVA test or the Mann-Whitney test. *, p < 0.05; **, p < 0.01; ***, p < 0.001.

To quantify the magnitude and expansion of CD8 and CD4 T cell responses in TBEV infected patients, we combined staining for CD38, which is expressed by activated T cells during acute and chronic infections [28], with staining for the intracellular marker Ki67 found exclusively in cycling or recently divided T cells [29]. CD4 and CD8 T cell gating strategy is found in S2 Fig. The patients’ levels of Ki67 and CD38 co-expressing activated CD8 T cells was 10-fold greater at 7 days than at 90 days after hospitalization and in comparison to levels in the healthy controls (Fig. 1A and 1B).

To evaluate general non-specific activation of CD8 T cells, known as bystander activation, in the course of infection we used an HLA-A2 tetramer refolded with a CMV-pp65 epitope peptide to identify CMV-specific cells. Activation was evaluated by the expression of Ki67, which was low in the CMV-pp65 tetramer-defined population and remained so throughout the course of infection in all tested donors (Fig. 1C and 1D). This finding suggests that bystander activation of CMV-specific CD8 T cells is not a major feature during acute TBEV infection.

To further characterize the CD8 T cell effector response defined by Ki67 and CD38 expression at the peak of expansion at day 7 after hospitalization, staining for CD45RA, PD-1, Bcl-2, CD127, CD27 and HLA-DR, together with perforin and granzyme B was performed (Fig. 1E). Expression of HLA-DR, PD-1, perforin and granzyme B was increased in activated CD8 T cells along with decreased expression of CD127, Bcl-2 and CD27 (Fig. 1F). We also measured the expression of CD127 (IL-7Ra), which has been shown to be downregulated on effector cells and then to be re-expressed on precursors of the memory pool [30], together with the homing marker CCR7 and CD45RA. Approximately 60% of the effector population consisted of a CD45RA^−^CCR7^−^CD127^−^ phenotype (Fig. 1G). These data showed that TBEV infection induced a robust CD8 T cell response that contracted to background levels over a period of 90 days post-hospitalization.

As CD4 T cells are an integral component in effective antiviral responses, we extended our study to CD4 T cell subsets. Elevated levels of CD38 and Ki67 co-expressing CD4 T cells were detected in TBEV-infected patients at day 7 after hospitalization, as compared to healthy controls (Fig. 1H and 1I). This CD38 and Ki67 co-expressing CD4 T cell population exhibited increased expression of HLA-DR, PD-1 and perforin together with low expression of Bcl-2, suggesting that activated CD4 T cells have effector properties at day 7 after hospitalization (S3A Fig.). The expression of CCR7, CD45RA and CD127 in the CD4 population was more variable in comparison to the corresponding population of CD8 T cells (S3B Fig.).

### Activated CD8 T cells are Eomes^+^T-bet^+^ at the peak of activation

**Figure 2:**
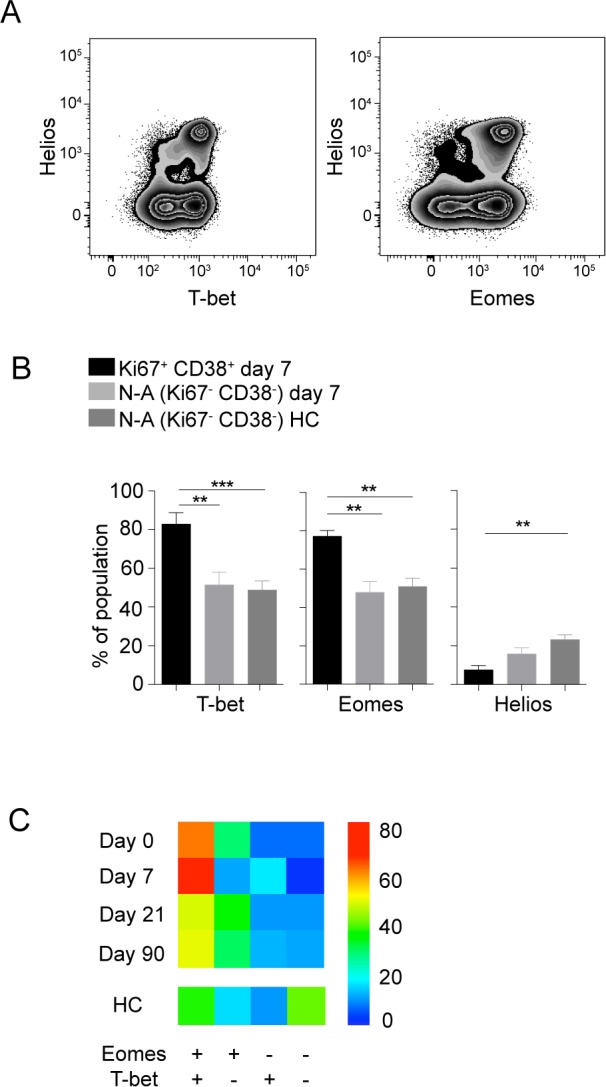
Transcriptional profile of activated CD8 T cells. (A) Flow plots show Helios, T-bet and Eomes stainings in CD8 T cells from one representative donor. (B) Bar plots show the 10–90th percentiles of T-bet, Eomes and Helios expression in the CD38 and Ki67 co-expressing CD8 T cell subset at day 7 after hospitalization (n = 10), and at day 7 after hospitalization in non-activated cells or in non-activated healthy controls (n = 16). (C) Heat map represents subset distribution of Eomes and T-bet within the CD38 and Ki67 co-expressing CD8 T cells (n = 10). Statistical analysis was performed using the Mann-Whitney test. *, p < 0.05; **, p < 0.01; ***, p < 0.001.

To further characterize the phenotype of the activated CD8 T cells, we assessed expression of transcription factors including T-bet, Eomes and the *Ikaros* family transcription factor Helios. The expression of T-bet and Eomes have been suggested to impact exhaustion and terminal differentiation of CD8 T cells [31], whereas Helios have been suggested as a marker of activation and proliferation in T cells [32]. The activated CD8 T cell population studied showed significantly increased expression of T-bet and Eomes, whereas Helios expression was lower, as compared to non-activated CD8 T cells in the same sample and to cells from healthy controls (Fig. 2A and 2B). The activated cells remained Helios negative throughout the course of infection and CD8 T cells expressing Helios expressed low levels of Ki67 and PD-1 throughout the infection (S4 Fig.). Cells with simultaneous expression of Eomes and T-bet constituted a dominant population of the activated CD8 T cells at day 7 after hospitalization (Fig. 2C). These data indicate that effector CD8 T cells at day 7 after hospitalization have a distinct Eomes^+^T-bet^+^ profile.

### TBEV-specific CD8 T cells are of a CD45RA^−^CD27^+^ phenotype with primarily mono-functional properties

**Figure 3:**
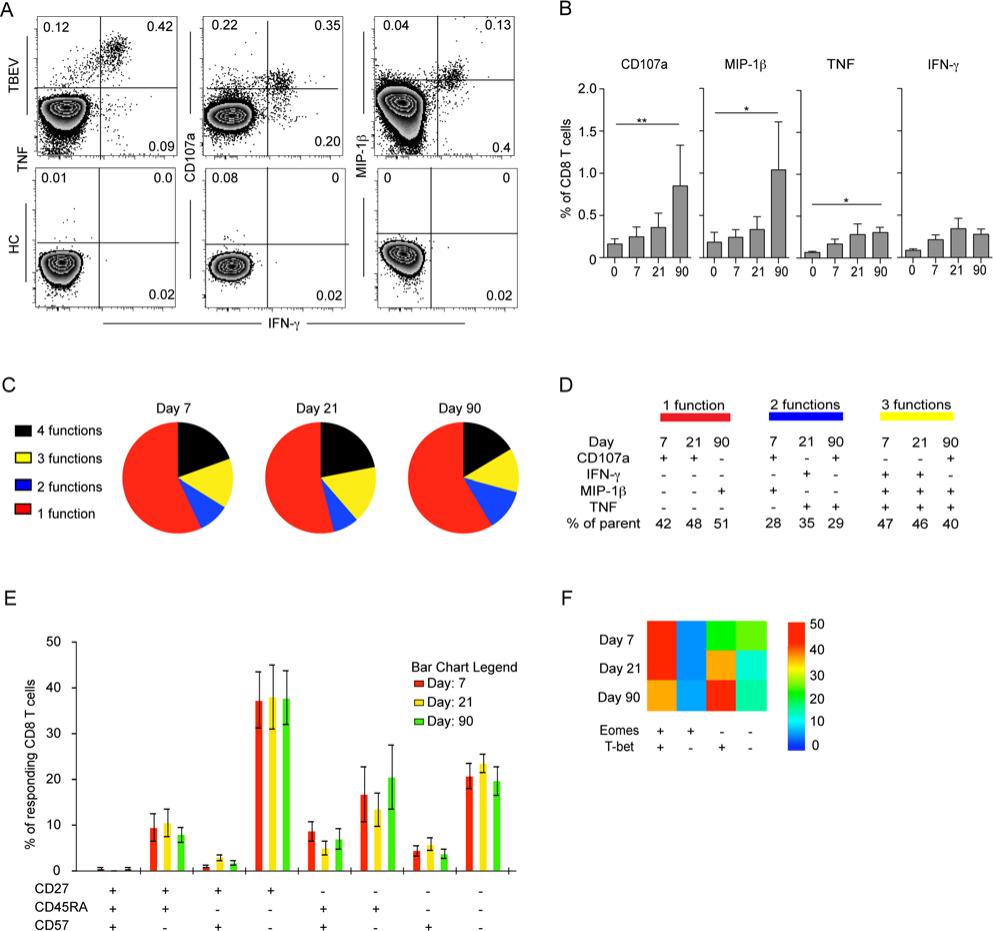
Functional profile of TBEV-specific effector and memory CD8 T cell responses. (A) PBMCs from infected subjects (n = 5) and healthy controls (n = 5) were stimulated for 6 hours with a pre-selected TBEV peptide pool in the presence of brefeldin A and monensin. Intracellular expression of MIP-1β, IFN-γ, and TNF, as well as the cell surface expression of CD107a, were assessed by flow cytometry and are shown as respresentative flow plots at day 21 after hospitalization. (B) Bar plots show the 10–90th percentiles of CD107a, MIP-1β, TNF and IFN-γ production in response to a pre-selected peptide pool at day 0, 7, 21 and 90 after hospitalization (n = 5). (C) Pie charts indicate the mean composition of the total response in CD8 T cells with regards to their capacity to express one, two, three or four functions at days 7, 21 and 90 after hospitalization. (D) Dominant polyfunctional profiles at day 7, 21 and 90 after hospitalization. The percentage of parent populations is indicated for each dominant population. (E) Bar chart represents the subset distribution of CD27, CD45RA and CD57 in cells responding with CD107a, MIP-1β, IFN-γ, or TNF after stimulus with a pre-selected peptide pool day 7, 21 and 90 after hospitalization. (F) Heat map represents subset distribution of Eomes and T-bet in cells responding with CD107a, MIP-1β, IFN-γ, or TNF after stimulus with the pre-selected peptide pool at day 0, 7, 21 and 90 after hospitalization. Statistical analysis was performed by using the non-parametric repeated measures ANOVA test. *, p < 0.05; **, p < 0.01; ***, p < 0.001.

CD8 T cells have a spectrum of functions to control viral infections. Here, we assessed degranulation (CD107a), cytokine expression (IFN-γ and TNF) and chemokine expression (MIP-1β) in TBEV-specific CD8 T cells over time in samples from five infected patients. We used a pool of potential TBEV peptide epitopes predicted by the NET-CTL algorithm (Table 1) to stimulate PBMCs *in vitro*, and studied the kinetics and functional profile of the responding cells during the course of infection. CD8 T cell responses were very low or undetectable on the day of hospitalization. The frequency of CD8 T cells expressing IFN-γ and TNF in response to the peptide pool peaked at day 21 after hospitalization, comprising approximately 0.5% of the total CD8 T cell population (Fig. 3A and 3B). CD107a together with MIP-1β peaked at day 90 with approximately 1.5% of the CD8 T cells (Fig. 3B). The response pattern was primarily mono-functional (>50%); however, approximately 5–10% of the responding cells exhibited a two-functional profile, 15% exhibited a three-functional profile, and around 20% of the cells displayed a four-functional profile. This pattern was sustained over time in the infected subjects (Fig. 3C). At the peak of the effector stage (days 7 and 21), CD107a mono-functional cells dominated the response, whereas MIP-1β-positive cells dominated the mono-functional response at day 90 after hospitalization (Fig. 3D). A more diverse pattern could be observed in the bi-functional cells, with CD107a-expessing and MIP-1β-producing cells dominating at day 7, IFN-γ and TNF producing cells dominating at day 21, and CD107a expressing and TNF producing cells dominating at day 90 after hospitalization (Fig. 3D). The triple functional cells produced mostly IFN-γ, MIP-1β and TNF at day 7 and 21, while CD107a, MIP-1β and TNF producing cells dominated at day 90 after hospitalization (Fig. 3D). These results indicate that the functional composition of TBEV specific CD8 T cells changes over time as they mature from an effector- to a memory-type response.

**Table 1:** TBEV peptides predicted by the NET-CTL algorithm.

**Nr**	**Position**	**Sequence**	**Combined Score NetCTL**	**SYFPEITHI score**	**Protein in TBEV**
A2–1	1984	ILLDNITTL	1.4354	31	NS3
A2–2	1207	TLLQAVFEL	1.3821	28	NS2A
A2–3	2242	KLAYFLLTL	1.3616	30	NS2B
A2–4	2190	MMLGTLVLL	1.3274	28	NS4A
A2–5	543	VLLKSLAGV	1.3195	30	E prot
A3–6	1879	CLNSKTFEK	1.6111	21	NS3
A3–7	3075	QLAATIMQK	1.5969	29	NS5
A3–8	2397	KVFFSAMVR	1.5791	25	NS4B
A3–9	1788	SIAARGHLY	1.4213	23	NS3
A3–10	576	KLKMKGLTY	1.4143	31	E prot
B7–11	2084	RPVWRDARM	1.7342	18	NS3
B7–12	2824	APTGSAASL	1.7063	25	NS5
B7–13	2469	LPLGHRLWL	1.5078	25	NS4B
B7–14	1113	RPVHDQGGL	1.4846	21	NS1
B7–15	354	CPTMGPATL	1.447	21	E prot

To further characterize cells responding to the predicted TBEV epitopes, we assessed the expression of CD45RA, CD27 and the senescence marker CD57 on the surface of TBEV-specific CD8 T cells. CD8 T cells responding to the peptide-pool with CD107a, IFN-γ, TNF or MIP-1β expression consisted mainly of a CD27^+^CD45RA^−^CD57^−^ phenotype (Fig. 3E). The transcription factor expression pattern in CD8 T cells responding to the peptide pool was similar to that of the Ki67^+^CD38^+^ activated CD8 T cells (Fig. 2C), with a dominant Eomes^+^T-bet^+^ profile comprising 50% of the responding cells at day 7 after hospitalization (Fig. 3F).

### CD8 T cells specific for an HLA-A2-restricted immunodominant epitope display a CD45RA^−^CCR7^−^PD-1^+^CD57^−^ phenotype in the effector stage of TBEV

**Figure 4:**
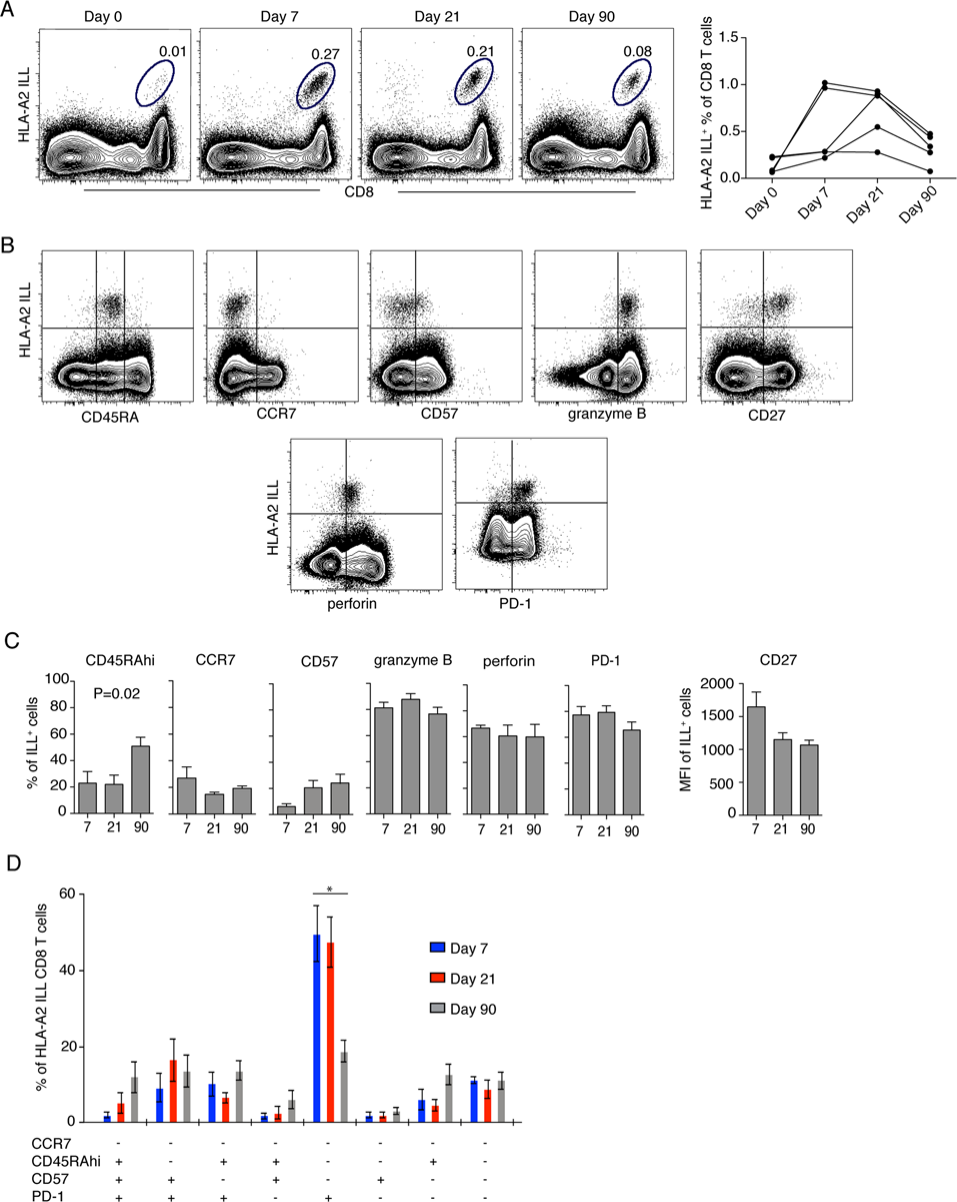
Effector and memory differentiation of TBEV-specific CD8 T cells. (A) Flow plots show HLA-A2 NS3 ILL^+^ CD8 T cells in one representative infected donor at day 0, 7, 21 and 90 after hospitalization (left panel), NS3 ILL-specific cells over time in five donors (right panel). (B) Flow plots representing the phenotype of TBEV-specific CD8 T cells. Plots are gated on total CD8 T cells at day 7 after hospitalization from one infected patient. (C) Results from the longitudinal phenotypic analysis of HLA-A2 NS3 ILL^+^ CD8 T cells present in the blood of five HLA-A2 patients. Bar plots show the median and 10–90th percentiles at day 7, 21 or 90 after hospitalization of CD45RA, CCR7, CD57, granzyme B, perforin and PD-1. CD27 is presented as mean fluorescence intensity (MFI). (D) Kinetics of HLA-A2 ILL-specific CD8 T cells. Bar chart represents the subset distribution of CCR7-, CD45RA-, CD57- and PD-1- expressing cells. Statistical analysis was performed using the non-parametric repeated measures ANOVA test. *, p < 0.05; **, p < 0.01; ***, p < 0.001.

Responses to the TBEV peptide pool indicated that the pool contained at least one epitope targeted by the CD8 T cells during acute TBEV infection. Single peptide stimulations identified one HLA-A2-restricted peptide (ILLDNITTL) in the NS3 protein, which induced cytokine responses in all tested A2^+^ donors. The NS3 ILL-specific HLA-A2 tetramer identified detectable frequencies of cells in five donors with up to 1% positive cells at day 7 and 21 after hospitalization, whereas tetramer-positive cells were barely detectable at the day 0 time point (Fig. 4A). Tetramer-positive CD8 T cells were further characterized for the expression of CD45RA, CCR7, CD57, granzyme B, perforin, PD-1, and CD27 (Fig. 4B and 4C). During the effector response at day 7 after hospitalization, the most prevalent phenotype of the TBEV-specific CD8 T cells was Tem, CD57^−^ and PD-1^+^, with this phenotype representing approximately 50% of the cells. This phenotype decreased and was less common at days 21 and 90 after hospitalization (Fig. 4D). These data demonstrate how the TBEV-specific CD8 T cell response goes through a Tem, CD57^−^PD-1^+^ character that contracts with the establishment of T cell memory.

### CD8 T cells specific for TBEV show a dominant Eomes^−^Ki67^+^T-bet^+^ effector phenotype that transition to an Eomes^−^Ki67^−^T-bet^+^ memory phenotype as the response matures

**Figure 5:**
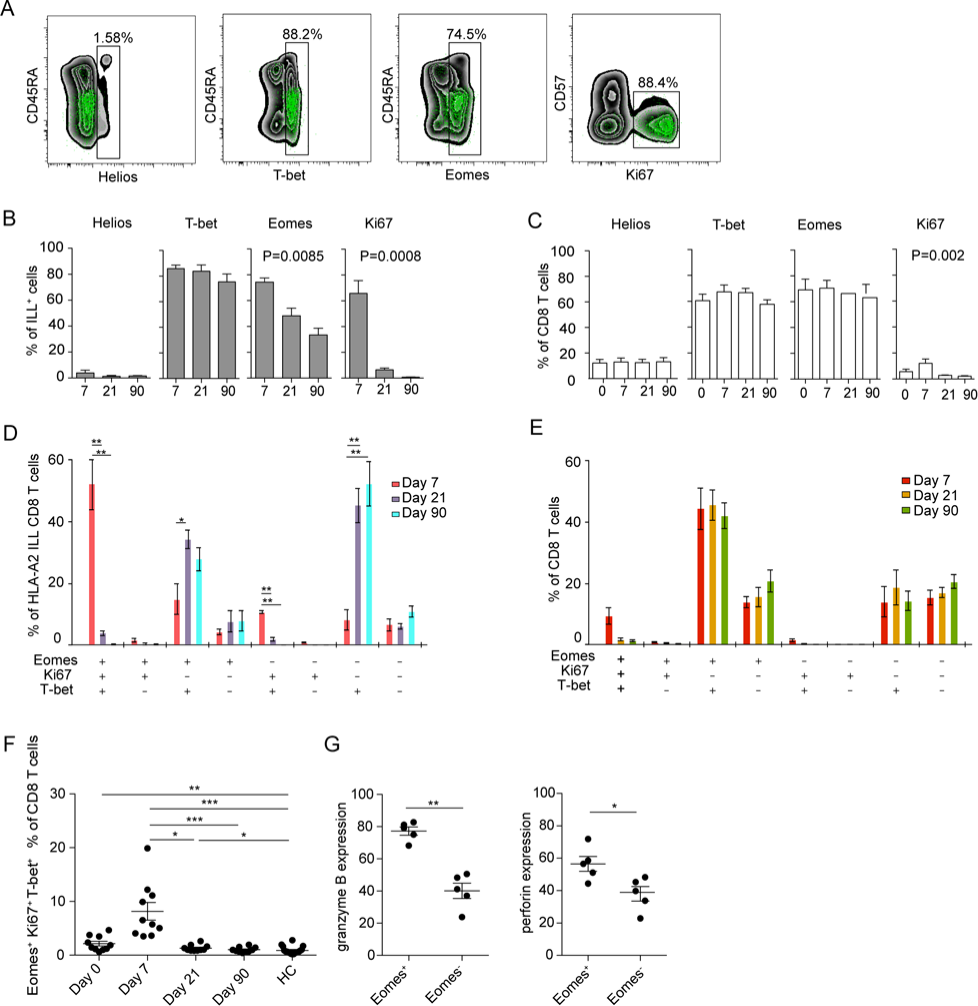
Transcription factor profile of TBEV-specific cells. (A) Plots are gated on total CD8 T cells (black background) or TBEV-specific (NS3 ILL) CD8 T cells (green dots). (B) Bar plots show the median and 10–90th percentiles of each marker in ILL^+^ specific cells from five donors. (C) Bar plots show the median and 10–90th percentiles of each marker in total CD8 T cells from five donors (D) Bar chart represents subset distribution of T-bet, Eomes and Ki67 in TBEV-ILL-specific cells. (E) Bar chart represents subset distribution of T-bet, Eomes and Ki67 in total CD8 T cells. (F) Median and 10–90th percentiles of Eomes^+^Ki67^+^T-bet^+^ total CD8 T cell subset at day 7, 21 or 90 after hospitalization in infected subjects (n = 10) together with healthy controls (n = 16). (G) Median and 10–90th percentiles of granzyme B and perforin in Eomes^+^ and Eomes^−^ total CD8 T cell subsets at day 7 after hospitalization. Statistical analysis was performed by using non-parametric repeated measures ANOVA test or Mann-Whitney test. *, p < 0.05; **, p < 0.01; ***, p < 0.001.

CD8 T cell populations specific for human polyomavirus BK virus, influenza virus, CMV, and EBV display specific patterns of T-bet and Eomes expression in healthy blood donors [33]. Since the activated (Ki67^+^CD38^+^) CD8 T cell population in TBE patients had increased expression of T-bet and Eomes along with low expression of Helios (Fig. 2B), we measured the expression of these transcription factors in TBEV-specific CD8 T cells. NS3 ILL-specific CD8 T cells expressed no or very low levels of Helios, whereas T-bet was highly expressed at all time-points (Fig. 5A and 5B). About 75% of the TBEV-specific CD8 T cells expressed Eomes at day 7 after hospitalization, declining over time to about 40% of the cells expressing Eomes at day 90 after hospitalization (Fig. 5B). Ki67 expression in NS3 ILL-specific CD8 T cells was high at day 7 after hospitalization, and declined over time, to become almost undetectable at day 90 after hospitalization (Fig. 5B). For total CD8 T cells, the expression levels of Helios was consistent around 10–15% at all time points (Fig. 5C). The expression patterns of T-bet and Eomes also remained stable at all time points, whereas Ki67 expression in total CD8 T-cells was at its highest level at day 7 after hospitalization at around 10–15%, and declined by day 21 after hospitalization (Fig. 5C). NS3 ILL-specific CD8 T cells thus have much less expression of Helios compared to the total CD8 T cell pool, along with a significant Eomes down regulation over time. The dominant phenotype of the TBEV-specific CD8 T cell population was Eomes^+^Ki67^+^T-bet^+^ at day 7, as found in 50% of the epitope-specific cells (Fig. 5D). This phenotype retracted to become almost undetectable by day 21 and 90 after hospitalization. Instead, an Eomes^−^Ki67^−^T-bet^+^ phenotype appeared by day 21 and 90 (Fig. 5D). The dominant phenotype in the total CD8 T cell pool was Eomes^+^Ki67^−^T-bet^+^ and this did not change over time (Fig. 5E). These results thus suggest that a set of three intracellular factors (Ki67, Eomes and T-bet) characterize and discriminate stages of the antigen-specific T cell response during acute TBEV infection.

We then also attempted to test the hypothesis that Ki67, Eomes, and T-bet could identify the total CD8 T cell effector responses in acute TBEV-infection. To this end, we analyzed this population in total CD8 T cells over time in ten TBEV infected donors. Co-expression of Eomes, Ki67 and T-bet peaked at day 7 after hospitalization in the infected study subjects, contracting to healthy control levels at day 21 after hospitalization (Fig. 5F). These levels were similar to Ki67 and CD38 positive CD8 T cells in Fig. 1B, and may represent an alternative way of describing activated CD8 T cells.

Eomes has been shown to play a role in induction of cytolytic properties in murine CD8 T cells [34]. With our observation that Eomes expression declines from 75% of the ILL-specific cells at day 7 after hospitalization to a lower percentage at day 21 (50%) and day 90 after hospitalization (35%) (Fig. 5B), we next aimed to further evaluate the role of Eomes and the global CD8 T cell cytotoxic potential at day 7 after hospitalization. We investigated the expression of granzyme B and perforin in Eomes^+^ and Eomes^−^ CD8 T cells. Eomes^+^ CD8 T cells expressed higher levels of both granzyme B and perforin than did Eomes^−^ cells (Fig. 5G), suggesting that Eomes expression was associated with the expression of cytolytic effector proteins.

## Discussion

Understanding T cell responses during viral infections is necessary for the successful design of antiviral treatments and vaccines. We here pursued a detailed analysis of the temporal dynamics, specificity, as well as functional and phenotypical characteristics of the CD8 T cell response to acute human TBEV-infection. Peripheral blood CD8 T cells were activated (determined by Ki67 and CD38 expression) in response to infection and expressed perforin, granzyme B, HLA-DR, PD-1, T-bet and Eomes together with low levels of Bcl-2 at day 7 after hospitalization, phenotypically defining these as effector cells. An immunodominant HLA-A2-restricted TBEV epitope was identified, and the corresponding HLA-tetramer defined TBEV-specific effector cells that predominantly displayed an Eomes^+^Ki67^+^T-bet^+^ effector phenotype at the peak of the response. The TBEV-specific CD8 T cells transitioned to an Eomes^−^Ki67^−^T-bet^+^ population as the infection resolved and memory was established. In summary, CD8 T cells responded to the virus and passed through an effector phase during acute TBEV-infection, prior to a gradual differentiation into memory cells with a distinct expression-pattern of transcription factors. The present results indicate that virus-specific effector CD8 T cells during acute TBE can be defined by the expression pattern of Eomes, Ki67, and T-bet within the global CD8 T cell compartment.

T-bet and Eomes are important in murine terminal effector and memory T cell development [23, 35] and cooperate by inducing the expression of IFNγ, granzyme B and perforin early in the activation process of murine CD8 T cells [23, 36, 37]. Although T-bet and Eomes cooperate in many respects, their expression is to some extent reciprocal. T-bet expression has been reported to be highest in early effector CD8 T cells in mice, but its expression progressively declines as memory cells form [38]. In contrast, the expression of Eomes is upregulated in early effector cells, and is sustained and increased during the effector to memory cell transition [35, 36]. Their expression pattern in TBEV-infection suggests that simultaneous upregulation is required to generate proper effector responses to control the infection in the acute stage. In human memory T cell subsets, the expression pattern of T-bet and Eomes may differ depending on the antigen. For instance, polyomavirus BK-specific cells display a T-bet intermediate and Eomes low phenotype. In the same donors, CMV-specific cells were high in both T-bet and Eomes, whereas influenza-specific cells were T-bet-high and Eomes-low [33]. The latter pattern is consistent with the phenotype of TBEV NS3 ILL-specific cells 90 days after hospitalization for TBEV infection (T-bet^high^, Eomes^low^). Thus, long-term human memory T cells specific for cleared infections such as influenza or TBEV may have a shared Eomes-low profile, which is distinct from T cells specific for persisting infections such as CMV.

Results from murine models indicate that Helios plays an important role in T cell development [39, 40]. Furthermore, Helios has been suggested as a marker of activation and proliferation in T cells, since Helios positive CD8 T cells are enriched for mature cells in humans and mice, and Helios become upregulated under *in vitro* stimulations of murine CD4 T effector cells [32]. In contrast, to date, very little is known about Helios in human CD8 T cells. In the present study, at the peak of TBEV infection, around 10–15% of the total CD8 T cells were Helios-positive. These cells were CD45RA^+/-^, CCR7^−^, Ki67^low^, CD57^int/-^, PD-1^−^, CD27^+/-^, CD45RA^hi^ and expressed perforin and granzyme B (S4 Fig.). With regards to previous publications, we initially speculated that the activated antigen-specific cells would express Helios. However, most Ki67 positive NS3 ILL-specific cells were negative for Helios.

The expression pattern of Eomes, Ki67 and T-bet was used to study the response longitudinally in CD8 T cells during TBEV infection. Approximately 5% of the CD8 T cells expressed a T-bet^+^Eomes^+^Ki67^+^ profile at day 7 after hospitalization. Cells with this phenotype contracted to healthy control levels at day 21 after hospitalization. Interestingly, this transcription factor co-expression-pattern was also observed in HLA-A2 tetramer-positive TBEV-specific cells. Therefore, Ki67 and CD38 co-expression describes the effector response in TBEV infection and the combination of T-bet, Eomes and Ki67 delineates the majority of antigen-specific cells in the acute stage of disease. Eomes has been suggested to play an important role in inducing lytic function in murine CD8 T cells [34] and consistent with this, we found that Eomes^+^ CD8 T cells express higher levels of both granzyme B and perforin than Eomes- CD8 T cells. In the case of T-bet, it has been shown that human CD8 T cells expressing high levels of T-bet rapidly can upregulate perforin upon stimulation with peptides [41].

Similar to the activation pattern of CD8 T cells, the HLA-A2-restricted NS3 ILL epitope-specific CD8 T cell response was absent at the day of hospitalization but appeared one week later. This indicates that the primary T cell response to TBEV infection occurs at this time and, hence, likely not in the first phase of infection; at least not in those patients which have a biphasic course of disease. In a YFV vaccine-based infectious model, the peak of viremia occurred at day 7 after immunization, and the peak of the CD8 T cell response was observed at day 15 after immunization [22]. However, TBEV RNA is usually not detected in plasma samples from patients during the neuroinvasive phase [42], but has been detected in sera and whole blood from patients during early stages of infection before the appearance of antibodies [43], and in brain tissue from patients dying from the disease [44]. We did not detect TBEV RNA at any time in plasma or cerebrospinal fluid (CSF) samples in our patient cohort; however, all the patients had seroconverted at the time of inclusion, and had entered the meningoencephalitic phase. Plausible reasons for the absence of detectable viral TBEV RNA would be that the primary viremia already had occurred and that the viral burden driving the CD8 T cell response was below the limit of our assay’s detection at the time of sampling. Alternatively, the virus may be located in other cells and tissues that produce and release viral antigen that stimulate the CD8 T cell response. Undetectable levels of viral RNA in blood with simultaneous presence of viral RNA in urine has previously been described in patients with Dengue fever [45], and after vaccination against YFV [46]. West Nile Virus (WNV) RNA was detected in the urine of patients with symptomatic WNV infection (neuroinvasive disease and fever) at a higher rate and load and for a longer time than in the plasma of these patients, whereas the detection rate of WNV RNA in urine was lower than in plasma in asymptomatic donors [47]. The mean number of days after the first symptom debut in our patient cohort was 15 days, indicating that the TBEV-specific T-cell response in peripheral blood appeared at about day 21 in the course of infection. However, it is not known what the CD8 T cell response looks like at the site of pathogenesis, i.e., the central nervous system, and if the TBEV-specific CD8 T cells that we observe in the periphery are representative of the population of CD8 T cells that is able to cross the blood-brain barrier. TBE pathogenesis is still largely unknown. TBEV has been shown to be present in brain tissue in most of the fatal cases [6], so direct infection of neurons may cause the neurological disease; however, in the majority of patients it is not possible to detect TBEV RNA in CSF [42]. There are also studies suggesting that the CD8 T cell response in CNS contributes to the pathogenesis in humans and mice [8, 9]. In this study, we were not able to draw any conclusions on relationships between disease severities and the phenotype or magnitude of TBEV-specific CD8 T cells, or virus replication in the CNS. Future studies with larger number of patients may help delineating the mechanisms and steps of the disease.

Activated CD8 T cells (Ki67^+^CD38^+^) were characterized by effector properties, such as increased expression of perforin and granzyme B, together with a Tem, CD127- profile. This phenotype has previously been studied in human yellow fever- and smallpox vaccine-models of acute viral infection [22]. Activated CD8 T cells were also observed in natural acute Hantavirus infection in humans, where up to 50% of the CD8 T cells have an activated profile one to two weeks after the symptom debut [48]. No or minimal bystander activation of CMV-specific memory CD8 T cells was detected during acute TBEV infection. This is in line with results by Miller *et al*., who showed that CMV-, EBV- and influenza virus-specific CD8 T cells did not contribute to the effector T cell response to YFV and smallpox vaccines [22]. Together, these findings support the notion that the majority of activated CD8 T cells in the present patient cohort were specific for TBEV antigens. With regard to the NS3 ILL-specific cells, the dominant phenotype observed at the peak of activation was Tem PD-1^+^CD27^high^, which decreased significantly to approximately 15% of the peak in the convalescent phase. TBEV-specific cells show a Tem, PD-1^+^CD57^−^ phenotype which is similar to what has been shown at the peak of the response to the YFV vaccine [20, 21]; however, after YFV vaccination, a CD45RA^+^CCR7^−^ late-stage effector cell (TemRA) phenotype was observed at the memory stage. It has been reported that TemRA cells may be involved in protective immunity against HIV, since HIV-specific T cells with this phenotype were associated with control of viremia [49, 50]. Therefore, memory may not be fully formed three months after infection with TBEV since such a population is detectable, but not dominant, in NS3 ILL-specific cells. Given that TBEV is cleared after the acute phase of infection, the TBEV-specific CD8 T cell population may eventually obtain a central memory-like phenotype [51]. The quality of a T cell response is probably important for the level of protection it provides to the host. CD8 T cells can provide a range of effector functions, which rarely are co-expressed in the same cell with the same kinetic pattern. The heterogeneity in expression of CD8 T cell effector functions has been described [52, 53], but is not well understood. Our results show that cells responding to a pool of pre-selected TBEV peptides predominantly displayed monofunctional characteristics.

In summary, the present results describe the phenotype and function of the CD8 T cell responses in acute TBEV-infection. In addition, based on the transcription factor expression profile in the TBEV-specific cells, Eomes, Ki67 and T-bet identifies cytolytic virus-specific CD8 T cells in the peak effector stage of acute TBEV infection. TBE is an emerging disease and is a growing health challenge in endemic parts, and with no antiviral drugs available. Indeed, the only effective protection against TBEV is vaccination. However, over the last few years vaccine failures have been reported, and it is also believed that a number of vaccine failures may have been overlooked due to difficulties in diagnosis, partly due to unusual antibody-kinetics in this patient group [54]. Taken together, these data may prove to be helpful for the future design of new therapeutic and immunotherapeutic treatment regimens as well as new options for vaccines to TBEV infection.

## Materials and Methods

### Ethics statement

All included patients and healthy individuals gave written informed consent to participate in the study and the Kaunas Regional Research Ethics Committee, Lithuania and the Regional Ethical Review Board in Stockholm, Sweden approved of the study.

### Study design and subjects

Blood samples and cerebrospinal fluid used were collected after written informed consent and with approval from the Kaunas Regional Research Ethics Committee, Lithuania and the Regional Ethical Review Board in Stockholm, Sweden. Peripheral blood was collected from twenty confirmed (IgM positive for TBEV in serum according to standard clinical diagnostic criteria) TBEV infected patients hospitalized at the Clinic of Infectious Diseases at Lithuanian University of Health Sciences Kaunas in Lithuania. PBMC were isolated from CPT tubes (BD Biosciences, San Jose, CA) and cryopreserved in 90% FCS and 10% DMSO for later analysis. Whole blood and plasma were collected and cryopreserved for later analysis.

### Antibodies for flow cytometry

T cell responses to TBEV were assessed using multi-color flow cytometry, and the monoclonal antibodies (mAbs) used were; anti-CD107a FITC, anti-CD4 Pacific Blue, anti-CD8 PerCP, anti-HLA-DR PerCP, anti-Ki67 FITC, anti-Ki67 Alexa Fluor 700, anti-Bcl2 PE, anti-CCR7 PE-Cy7, anti-MIP-1β PE, anti-CD14 BD horizon V500, anti-CD19 BD horizon V500, anti-perforin FITC and anti-granzyme B APC, anti-granzyme B PE-CF594 all from BD Biosciences (San Jose, CA). Anti-CD45RA APC-Cy7, anti TNF pacific blue, anti-IFN-γ Brilliant Violet 570, anti-CD27 Brilliant Violet 785, anti-CD27 Brilliant Violet 421, anti-Helios Pacific Blue, anti-T-bet Alexa Fluor 488, anti-T-bet PE-Cy7, anti-CCR7 Brilliant Violet 785, anti-CD279 Brilliant Violet 711, anti-CD27 biotin and anti-CD127 Brilliant Violet 570 were all from Biolegend (San Diego, CA). Anti-CD38 Alexa Fluor 700, anti-CD38 eFluor 650, anti-CD127 Alexa Fluor 780, anti-PD-1 (CD279) PE, anti-Eomes eFluor 660 and IgM eFluor 650 were all from eBioscience (San Diego, CA). Anti-CD4 Qdot 605, anti-CD8 Qdot705, anti-CD8 Qdot 605, Streptavidin-Qdot 585, anti-CD57 pure and Aqua Live/Dead were all from Invitrogen (Carlsbad, CA). Anti-CD3 ECD, anti-CD3 PE-Cy5, HLA-A2 CMV pp65 tetramer in PE and anti-CD56 ECD were from Beckman Coulter (Brea, CA). HLA-A2 ILLDNITTL tetramer in PE was kindly provided by the NIH Tetramer core facility.

### Flow cytometry

For phenotypic analysis of cells, PBMCs were incubated for 30 minutes 4°C in the dark, with surface mAbs, followed by washing with PBS. For the CD107a staining, the CD107a antibody was present during the 6 hours stimulation, and then additional CD107a antibody was added together with the surface mAbs for 30 minutes incubation at 4°C in the dark. Cells were fixed and permeabilized with fix/perm (eBioscience) for 30 minutes at 4°C in the dark. Cells were then washed and stained with intracellular mAbs. Samples were acquired on a BD LSRFortessa instrument (BD Biosciences) and analyzed using FlowJo software version 9.4 (Tree Star, Ashland, OR), and SPICE 5.3 software provided by Dr. M. Roederer (National Institutes of Health, Bethesda, MD) [55].

### Epitope search and synthetic peptides

Candidate epitopes of HLA-A2, -A3 or-B7 super-types were predicted using the NetCTL search engine (version 1.2) [56]. The HLA class I epitope predictions were performed on polyprotein consisting of a TBE EK-328 strain available in the Flavitrack database at http://carnot.utmb.edu/flavitrack. 15 peptides were predicted, and the 5 top scoring peptides in each HLA super-type were selected for synthesis. Peptides were synthesized by standard 9-fluorenyl-methyloxycarbonyl (FMOC) chemistry, purified to 70% purity by reverse-phase high-performance liquid chromatography and validated by mass spectrometry (JPT Peptide Technologies, Berlin, Germany).

### In vitro functional assays

PBMCs were rested in RPMI 1640 medium containing 10% FCS, 2 mM L-glutamine, 1% penicillin and streptomycin (Invitrogen) overnight at 37°C. Cells were stimulated with 10 μg peptides for 6 hours in 96-well round bottom plates in the presence of Brefeldin A (Sigma-Aldrich, St. Louis, MO), monensin (BD Biosciences) and purified anti-CD28/CD49d (1 μl/ml) (BD Bioscienses). Staining, flow cytometry and analyses were performed as described above.

### HLA class I typing

Patient and control genomic DNA was isolated from whole blood using DNeasy kit (QIAGEN). HLA typing was performed using a multiplexed reverse sequence-specific oligonucleotide probe method (LABType SSO; One Lambda), according to the manufacturer’s instructions.

### Detection of TBEV-specific CD8 T cells

For detection of TBEV-specific CD8 T cells were identified in peripheral blood by staining PBMCs with HLA-A2 NS3 ILLDNITTL tetramer for 15 minutes at 4°C in the dark, prior to the addition of surface mAbs. Cells were fixed and permeabilized with fix/perm (eBioscience) for 30 minutes at 4°C in the dark before the addition of intracellular mAbs. Samples were acquired on a BD LSRFortessa instrument (BD Biosciences)

### Quantitative RT-PCR

Detection of TBEV RNA was assessed as previously described [57] with these minor changes: RT-PCR was performed with TaqMan Fast Virus 1-step mastermix (Applied Biosystem, Life Technologies) using the StepOne RT-PCR system (Life Technologies) according to the manufacturer’s instruction. To ensure adequate RNA extraction from the samples, human B-actin (Applied Biosystems, Life Technologies) was assayed in parallel as an endogenous control.

### Serology

All sera were analyzed by using the Siemens Enzygnost TBE IgG assay (Siemens Healthcare Diagnostics, Erlangen, Germany), and the sera isolated from blood draws at the first two time points of each patient were also analyzed by Immunozym FSM IgM assay (Progen Biotechnik GmbH, Heidelberg, Germany). These analyses were performed according to the manufacturer’s instructions by using a combination of the Freedom EvoClinical pipetting platform (Tecan Group Ltd, Männerdorf, Switzerland) and the BEP III system (Siemens Healthcare Diagnostics, Erlangen, Germany).

### Statistical analysis

Analyses were performed using GraphPad Prism software 5.0 for MacOSX (GraphPad Software, La Jolla, CA). Data were analyzed by non-parametric repeated measures ANOVA test or Mann-Whitney test P values < 0.05 were considered statistically significant.

## Supporting Information

S1 Table Study cohort and characteristics. (DOCX)

S1 Fig IgG in U/ml blood over time in TBE infected patients (n = 20). (TIF)

S2 Fig Gating strategy for CD4 and CD8 T cells. (TIF)

S3 Fig CD4 T cells in TBE infection. (A) Bar plots show the 10–90th percentiles of HLA-DR, Bcl-2, PD-1, perforin and granzyme B expression together with CD27 in terms of mean fluorescence intensity in CD38 and Ki67 co-expressing CD4 T cell subset at day 7 after hospitalization, non-activated Ki67^−^CD38^−^ (N-A) cells at day 7 after hospitalization or in non-activated healthy controls (N-A HC). (B) Bar chart represents the subset distribution of CCR7, CD45RA and CD127 (IL7Rα) in CD38 and Ki67 co-expressing cells at day 7 after hospitalization. Statistical analysis was performed using non-parametric repeated measures ANOVA test or the Mann-Whitney test. *, p < 0.05; **, p < 0.01; ***, p < 0.001.
(TIF)

S4 Fig Helios expression in CD8 T cells. (A) Flow plots representing the phenotype of Helios^+^ CD8 T cells from one representative donor at day 7 after hospitalization. Plots are gated on total CD8 T cells (black background), Helios^+^ CD8 T cells (red dots) or Helios^+^ TBEV-specific (A2-NS3) CD8 T cells (green dots). (B) Box and whisker plots show the median and 10–90th percentiles at day 0, 7, 21 or 90 after hospitalization of CD45RA, CCR7, Ki67, CD57, perforin, granzyme B, PD-1 and CD27 in Helios^+^ CD8 T cells from five donors.
(TIF)
