# Supplementary material for: Specificity and Dynamics of Effector and Memory CD8 T Cell Responses in Human Tick-Borne Encephalitis Virus Infection
Source: PLoS Pathog. 2015 Jan 22;11(1):e1004622. doi: 10.1371/journal.ppat.1004622 (PMC4303297; doi:10.1371/journal.ppat.1004622)
Supplement: S1 Table — (DOCX) [file ppat.1004622.s001.docx]

Supplemental Table 1. Study cohort and characteristics

| Patient | Age | Sex | Living area | TBE form | Biphasic course | Corticosteroid  Treatment | IgM  Day 0 | IgM Day 7 |
| --- | --- | --- | --- | --- | --- | --- | --- | --- |
| 1 | 48 | M | Rural | Mild | Yes | No | Pos | Pos |
| 2 | 58 | F | Rural | Moderate | Yes | Yes | Pos | Pos |
| 3 | 33 | M | Rural | Mild | No | No | Pos | Pos |
| 4 | 58 | F | Rural | Moderate | Yes | No | Pos | Pos |
| 5 | 54 | F | Rural | Mild | Yes | No | Pos | Pos |
| 6 | 54 | M | Rural | Mild | Yes | No | Pos | Pos |
| 7 | 31 | M | Rural | Mild | Yes | No | Pos | Pos |
| 8 | 54 | F | Rural | Moderate | No | Yes | Pos | Pos |
| 9 | 47 | M | City | Mild | Yes | Yes | Pos | Pos |
| 10 | 22 | F | City | Moderate | Yes | No | Pos | Pos |
| 11 | 63 | M | Rural | Mild | Yes | No | Pos | Pos |
| 12 | 57 | M | City | Mild | No | No | Pos | Pos |
| 13 | 37 | M | Rural | Mild | Yes | No | Pos | Pos |
| 14 | 26 | M | Rural | Mild | Yes | No | Pos | Pos |
| 15 | 65 | F | City | Mild | Yes | No | Pos | Pos |
| 16 | 54 | F | City | Mild | Yes | No | Pos | Pos |
| 17 | 46 | F | City | Mild | Yes | No | Pos | Pos |
| 18 | 58 | F | City | Mild | Yes | No | Pos | Pos |
| 19 | 56 | M | City | Mild | Yes | No | Pos | Pos |
| 20 | 43 | F | City | Mild | No | No | Pos | Pos |

Male (M), Female (F), Positive (Pos)
