# Supplementary figures and images for: Specificity and Dynamics of Effector and Memory CD8 T Cell Responses in Human Tick-Borne Encephalitis Virus Infection
Source: PLoS Pathog. 2015 Jan 22;11(1):e1004622. doi: 10.1371/journal.ppat.1004622 (PMC4303297; doi:10.1371/journal.ppat.1004622)

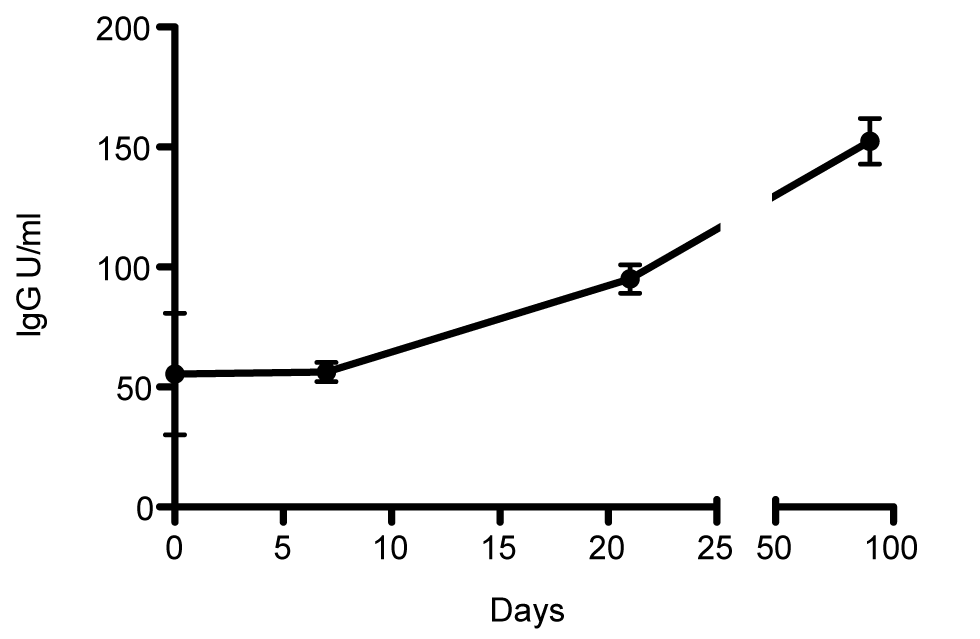

Supplement: S1 Fig — (TIF) [file ppat.1004622.s002.tif]

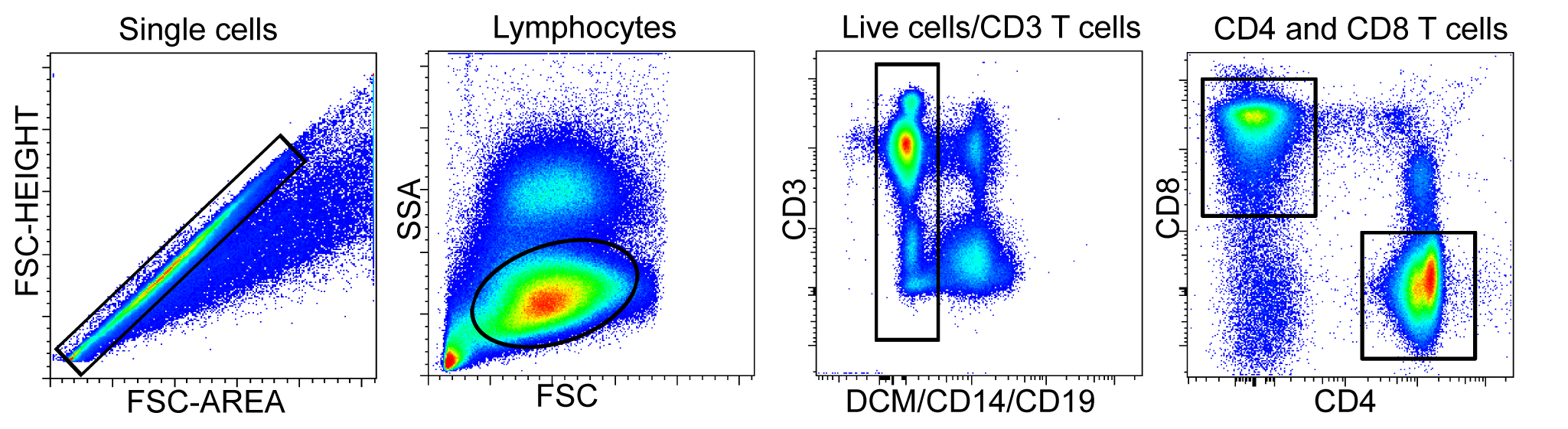

Supplement: S2 Fig — (TIF) [file ppat.1004622.s003.tif]

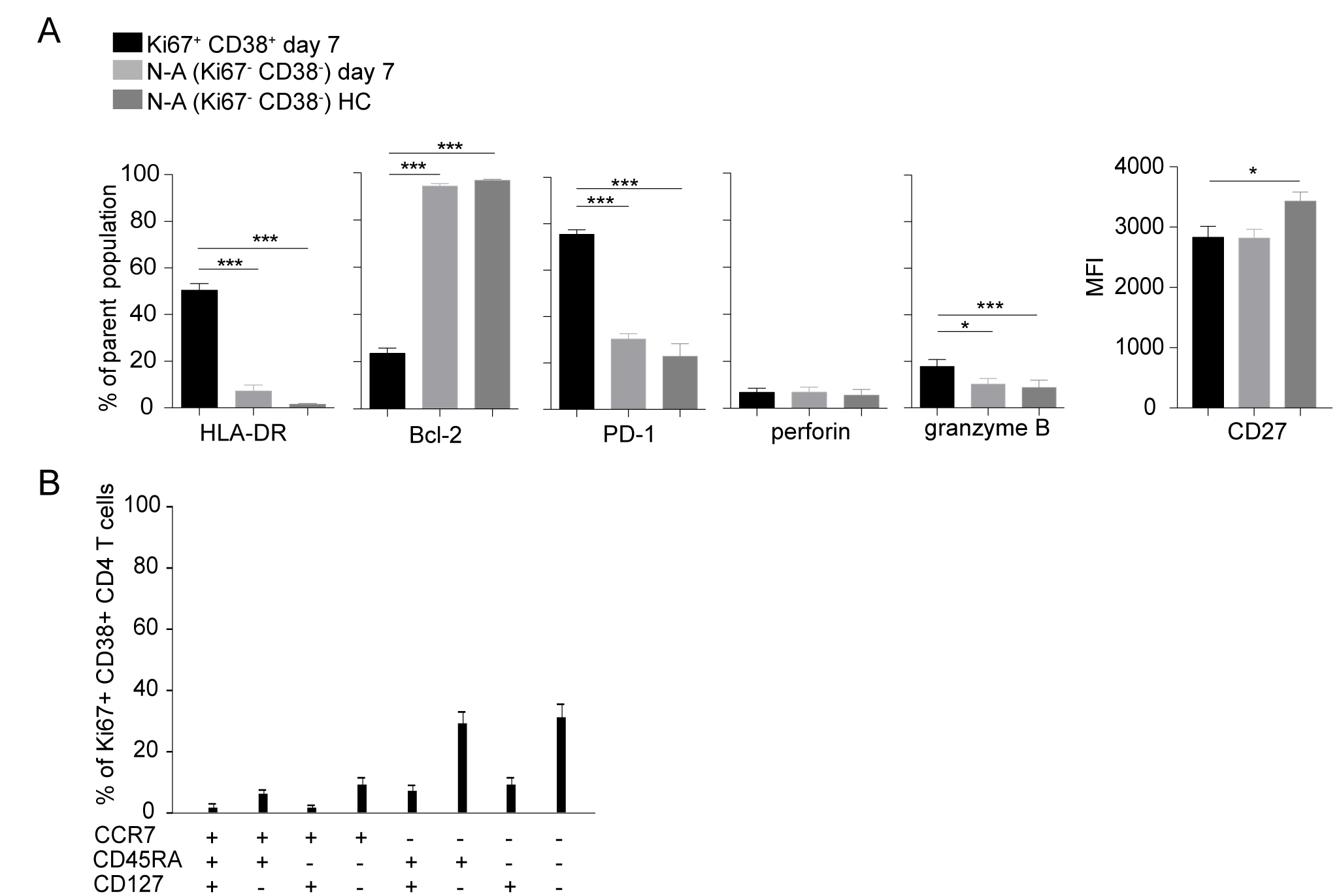

Supplement: S3 Fig — (A) Bar plots show the 10–90th percentiles of HLA-DR, Bcl-2, PD-1, perforin and granzyme B expression together with CD27 in terms of mean fluorescence intensity in CD38 and Ki67 co-expressing CD4 T cell subset at day 7 after hospitalization, non-activated Ki67−CD38− (N-A) cells at day 7 after hospitalization or in non-activated healthy controls (N-A HC). (B) Bar chart represents the subset distribution of CCR7, CD45RA and CD127 (IL7Rα) in CD38 and Ki67 co-expressing cells at day 7 after hospitalization. Statistical analysis was performed using non-parametric repeated measures ANOVA test or the Mann-Whitney test. *, p < 0.05; **, p < 0.01; ***, p < 0.001. (TIF) [file ppat.1004622.s004.tif]

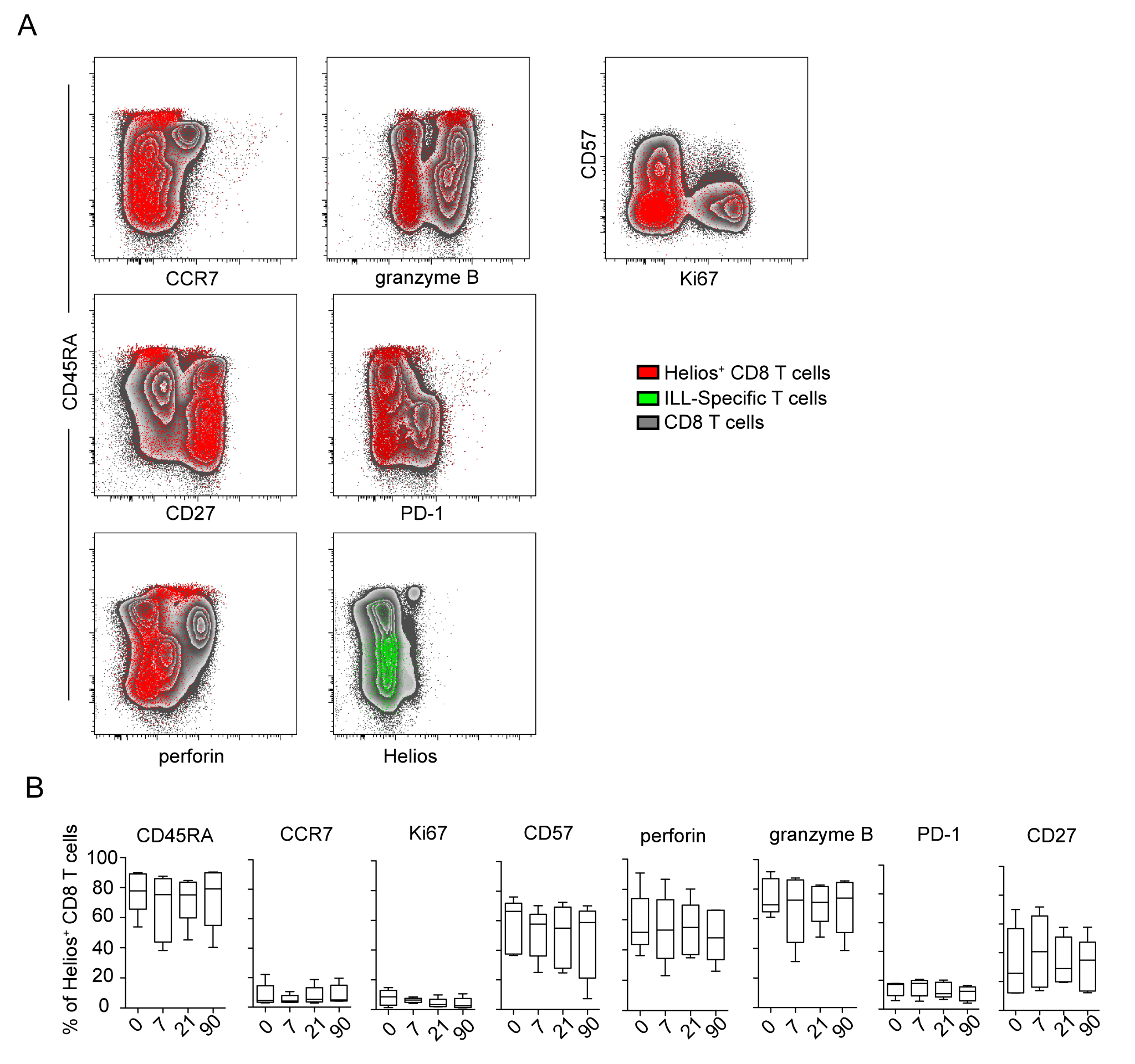

Supplement: S4 Fig — (A) Flow plots representing the phenotype of Helios+ CD8 T cells from one representative donor at day 7 after hospitalization. Plots are gated on total CD8 T cells (black background), Helios+ CD8 T cells (red dots) or Helios+ TBEV-specific (A2-NS3) CD8 T cells (green dots). (B) Box and whisker plots show the median and 10–90th percentiles at day 0, 7, 21 or 90 after hospitalization of CD45RA, CCR7, Ki67, CD57, perforin, granzyme B, PD-1 and CD27 in Helios+ CD8 T cells from five donors. (TIF) [file ppat.1004622.s005.tif]
